# Local stakeholder’s viewpoint on Vlassov’s contribution

**DOI:** 10.1093/eurpub/ckad210

**Published:** 2024-02-05

**Authors:** Charlotte Marchandise

**Affiliations:** EUPHA, Utrecht, The Netherlands

When the COVID pandemic broke out, I held the position of Deputy Mayor for Health in Rennes, France, and was also the President of the French WHO Healthy Cities Network, as well as the Chair of the European Political Vision group within this network. Additionally, I consulted for the WHO on Health in All Policies strategies. Actively involved in a political movement advocating for improved democracy in France, I championed our Mental Health motto ‘Nothing for us, without us’ in local and national policies, campaigning for greater ethics and transparency. I felt it was crucial, as 64% of French men and women were skeptical about the functioning of democracy, and 82% believed that political leaders disregarded their opinion.^1^

In my roles, I operated at both international and local levels, collaborating with scientists and citizens. As public health professionals, we recognized the pandemic’s potential threat and had included this in our network’s strategy. Aware of frameworks like the International Health Regulations, which were surprisingly underutilized in France, we worked closely with the National Public Health School. Given France’s notable vaccine hesitancy,^2^ we also focused on public acceptance and participation in Health decisions.

Indeed, the pandemic revealed numerous mistakes. As Vasiliy Vlassov highlighted, many decisions were flawed. While I will take his point: ‘the shock and awe of the first months of the pandemic excuse many mistakes’, I also believe France’s first mistake was the government’s failure to admit their lack of knowledge, leading to misleading strong affirmations about health measures and changing them without explaining the path of the decision. They claimed that ‘masks do not work’, then made them mandatory without a blink, and without an explanation if not an apology. The media gave ample coverage to figures like Didier Raoult, promoting unproven COVID treatments—hydroxychloroquine and pastis (an alcohol beverage from the south of France). People were fined for walking outside, and we are still paying the consequences on mental health, especially among the young.

In our Healthy Cities Network, existing partnerships enabled us to provide PPE, food aid to vulnerable groups, and psychological support for health workers and citizens. We coordinated actions, disseminated clear information and exchanged best practices and challenges with other cities, aiming to learn from our collective experiences; best practices as well as mistakes, ours and others’.

Despite our efforts, we struggled to gain national recognition. As local actors, as a network, and with our partners in Public Health networks, we tried to share our field knowledge with the national government, our lessons learned with the thousands of cities that do not belong to the network and do not benefit from expertise.

We failed too.

Our failure lay not in disregarding public health principles, equity, or transparency, but in advocacy. It should have been our moment to spotlight prevention and health promotion. Yet, we remained in the background, unable to amplify our voice. The public’s adherence to basic preventive measures like hand washing waned, and we missed the opportunity to leverage the crisis for better future preparedness. People stopped washing hands on a regular basis, there is no soap again in our schools, and coughing people do not wear masks in public transportation.

Moreover, this has exacerbated a diminishing trust in policymakers, indicating that we are not just facing a health crisis but also a crisis in democracy. I firmly believe that public health offers an exceptional avenue to unite people and tackle broader challenges, including the pressing issue of climate change.

In response to Vasiliy Vlassov’s invitation to detect and discuss the mistakes, ‘the first and necessary step to not to repeat them’, I suggest we expand the discussion to other actors. Our forthcoming memorandum of interest with WHO includes plans for closer collaboration with regions and cities, and we can harness the insights gained from this crisis. The upcoming election of new EU Members of Parliament is also a golden opportunity to amplify our message and influence policy. By expanding this dialogue, we aim to foster a more resilient and informed approach to public health, advocating for strategies that are not only effective but also equitable and transparent and adapted to local level, where health is shaped through all its determinants.

*Conflicts of interest*: None declared.
